# Elevated AIP is associated with the prevalence of MAFLD in the US adults: evidence from NHANES 2017–2018

**DOI:** 10.3389/fendo.2024.1405828

**Published:** 2024-05-14

**Authors:** Yan Chen, Chuan Lu, Haonan Ju, Qingzhi Zhou, Xin Zhao

**Affiliations:** Department of Cardiology, The Second Hospital of Dalian Medical University, Dalian, China

**Keywords:** metabolic syndrome, atherogenic index of plasma(AIP), hepatic steatosis, metabolic associated fatty liver disease (MAFLD), restricted cubic spline (RCS), NHANES

## Abstract

**Background:**

Atherogenic Index of plasma (AIP) is closely related to metabolic abnormalities. But as of now, there is no definitive conclusion on the dose-response relationship pattern between AIP and metabolic associated fatty liver disease (MAFLD).

**Objective:**

The objective of this study was to provide a fresh insight for understanding the intrinsic link between AIP and the prevalence of MAFLD by exploring the dose-response pattern between AIP and MAFLD.

**Methods:**

A total of 9254 participants received the survey and 1090 participants were finally included according to the screening criteria. To evaluate the association between AIP and the prevalence of MAFLD based on weighted multivariate logistic regression. Sensitivity analysis of the association between AIP and MAFLD was performed using propensity score matching (PSM). Restrictive cubic splines (RCS) were used to identify patterns of dose-response relationships between AIP and MAFLD, and receiver operator characteristic (ROC) curves were used to evaluate the predictive ability of AIP and traditional lipid parameters for MAFLD.

**Results:**

In this study, a total of 563 participants were found to have MAFLD. The results of weighted multivariate logistic regression analysis demonstrated that, after adjusting for sex and age, participants in the highest quartile (Q4) of AIP had a significantly increased risk of developing MAFLD compared to those in the lowest quartile (Q1) (Model 2: OR = 9.03, 95% CI 4.75–17.17). A similar trend was observed in the fully adjusted model (Model 3: OR = 3.85, 95% CI 1.55–9.52). The RCS analysis revealed a linear dose-response association between AIP and MAFLD(*P* for crude non-linearity = 0.087). This association remained significant after accounting for potential confounding variables(*P* for adjusted non-linearity = 0.663). The ROC curve results suggest that AIP performs better than traditional lipid indicators in predicting MAFLD (AUC = 0.732, 95%CI 0.705–0.758).

**Conclusion:**

A linear dose-response relationship exists between AIP and MAFLD, suggesting that as AIP increases, so does the risk of developing MAFLD.

## Introduction

In recent years, more and more attention has been paid to MAFLD characterized by hepatic steatosis and metabolic abnormalities (1, 2). According to statistics, the combined prevalence of MAFLD is 39.22% (3), especially in economically developed countries and regions. With the systematic definition of MAFLD diagnostic criteria (1), there is increasing recognition of the important role of metabolic abnormalities in such diseases. Unlike NAFLD, MAFLD is not a diagnosis of exclusion, and its definition identifies more patients with metabolic abnormalities and hepatic steatosis, thus having greater clinical applicability (1, 4, 5). Existing studies have indicated that MAFLD is superior to non-alcoholic fatty liver disease(NAFLD) in terms of identifying cardiovascular adverse events, risk of liver disease progression, and risk of all-cause mortality (4, 6, 7). This advantage might be attributed to the frequent presence of metabolic abnormalities observed among patients with MAFLD (8). With the growing prevalence of obesity and diabetes worldwide, the trend of the MAFLD pandemic may also worsen in the future (9, 10). Therefore, accurately identifying underlying MAFLD patients is crucial for improving their prognosis.

AIP is a novel lipid marker proposed by Dobiásová. It is obtained by performing logarithmic transformation on the ratios of triglyceride (TG) to high-density lipoprotein cholesterol (HDL-C) (11). AIP demonstrates an inverse relationship with lipoprotein particle size, leading to its recognition as a dependable proxy for small dense low-density lipoprotein cholesterol (sd-LDL-C) (11). Recent studies have shown that AIP outperforms traditional lipid markers in predicting the risk of cardiovascular disease (12–16). Interestingly, AIP is not only strongly associated with atherosclerosis, but also reflects the severity of insulin resistance(IR) in humans (17). Previous findings have shown that AIP is strongly associated with metabolic diseases, such as hypertension, diabetes, obesity, and atherosclerotic cardiovascular disease (15, 18–20). In view of the close association between AIP and indicators of human metabolic status (21), it is essential to investigate the potential connection between AIP and MAFLD to better find a convenient and easily accessible index for screening patients with MAFLD. Previous studies have verified the association between AIP and the prevalence of MAFLD among Chinese and Iranian individuals (22, 23). However, studies assessing the association between AIP and MAFLD prevalence among the American population remain scarce, and there is currently no research exploring the dose-response relationship between AIP and MAFLD prevalence.

Therefore, utilizing a cross-sectional analysis of NHANES (2017–2018) data, this study endeavors to illuminate the association between AIP and MAFLD prevalence among the general population in the United States. Furthermore, it explores the potential dose-response relationship patterns between AIP and MAFLD, offering a fresh perspective for assessing their association.

## Methods

### Study population

**Figure 1:**
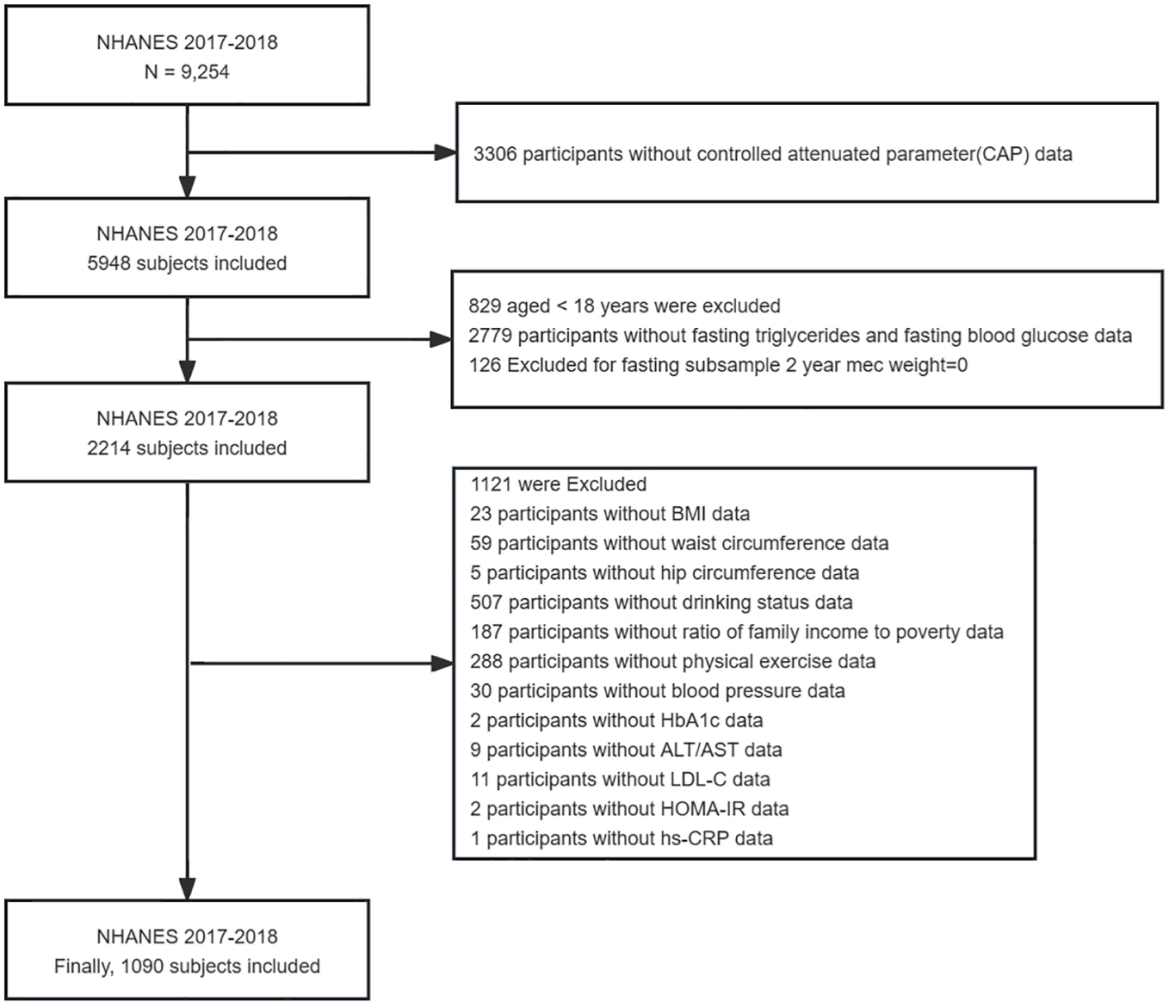
Flow chart of participants selection from the NHANES 2017–2018.

The NHANES, which commenced in the early 1960s and underwent a transformation in 1999, is a carefully planned study that assesses the comprehensive health and nutritional well-being of American adults and children (24). It has evolved into a continuous endeavor with a dynamic focus on diverse health and nutrition metrics to cater to evolving necessities. Each year, this survey meticulously examines a representative sample of approximately 5,000 individuals from across the nation. Data was gathered through face-to-face interviews and extensive health checks at mobile centers, employing a complex sampling method that ensured a representative cross-section of the population. Specifically, the study utilized information from the NHANES 2017–2018, encompassing 9,254 individuals. However, after rigorous screening, only 1,090 participants were deemed suitable for the analytical purposes of the research. The National Center for Health Statistics(NCHS) Research Ethics Review Committee has approved the survey and all participants have signed an informed consent form. The detailed screening process for selecting the final participants in this study is illustrated in **Figure 1**.

### Covariates

Covariates used in this study mainly included sex, age, race, education levels, economic status, BMI, smoking habits, alcohol consumption, waist and hip circumference, physical activity status, as well as health conditions like hypertension, diabetes, and hyperlipidemia. Economic status is gauged using the family income-to-poverty ratio (PIR), with classifications of < 1.0, 1.0–3.0, and > 3.0. The Body Mass Index (BMI) is calculated by dividing the weight in kilograms by the squared height in meters. Subsequently, it is classified into three categories: normal (BMI less than 25 kg/m^2^ overweight (BMI ranging from 25 to less than 30 kg/m^2^ and obese (BMI of 30 kg/m^2^ or higher). Participants were divided into never smokers, former smokers, and current smokers based on their smoking history and current smoking status (25). Drinking status is determined based on specific criteria: heavy drinking is defined as consuming three or more drinks per day for women, four or more drinks per day for men, or engaging in binge drinking on at least five days per month. Moderate drinking is characterized by having two or more drinks per day for females, three or more drinks per day for males, or binge drinking on at least two days per month. Mild drinking is designated for those who do not meet the criteria for heavy or moderate drinking, while never drinking refers to individuals who have consumed less than twelve drinks in their lifetime (26). The data on the use of lipid-lowering drugs by participants was obtained from questionnaire survey. The lipid-lowering drugs in this study mainly included drugs that have a significant impact on TG and HDL-C, such as statins, fibrates, and ezetimibe. Physical activity status was divided into groups according to whether moderate intensity exercise was performed for 150 minutes or more in a week (27). Moderate-intensity exercise was defined as having MET values between 3 and 6, and since NHANES designed questions in the PAQ questionnaire all had MET > 3, these items were considered at least equivalent to moderate-intensity exercise. The investigators asked the participants according to the “vigorous/moderate recreational activity”, “vigorous/moderate recreational activity”, and “walk or bicycle” in the PAQ questionnaire, and if the participants answered “YES” according to the description of the question, they were further asked about the number of times they performed this exercise per week and the time of each exercise.To calculate their total weekly exercise time, simply multiply the frequency of weekly workouts by the length of each session. Finally, we summed the exercise time for all items to calculate the total time participants spent performing moderate intensity exercise for one week. Exercise time at Vigorous intensity was finally multiplied by 2 to convert to exercise time at Moderate intensity. Detailed questionnaire content is available in the NHANES website. Hypertension is diagnosed based on several factors, including a self-reported history of the condition, currently taking medication to lower blood pressure, or exhibiting an average systolic blood pressure that is 140 mmHg or above, and/or a diastolic blood pressure averaging 90 mmHg or more. The diagnostic criteria for diabetes mellitus are as follows: a self-reported physician diagnosis, fasting glucose levels of 7.0 mmol/L or above, glycosylated hemoglobin (HbA1c) levels of 6.5% or higher, and/or the current use of diabetes medications. Hyperlipidemia is determined by assessing various parameters, including LDL-C levels of 130 mg/dL or higher (equivalent to 3.37 mmol/L or above), total cholesterol levels of 200 mg/dL or higher (5.18 mmol/L or above), TG levels of 150 mg/dL or higher (1.7 mmol/L or above), and HDL-C levels below 40 mg/dL for men (less than 1.04 mmol/L) or below 50 mg/dL for women (less than 1.30 mmol/L). Additionally, the use of lipid-lowering medications is also considered in the determination of hyperlipidemia (28). The laboratory test measures include HbA1c, fasting glucose, hs-CRP, fasting TG, total cholesterol (TC), low-density lipoprotein cholesterol (LDL-C), HDL-C, ALT, AST, and HOMA-IR. HOMA-IR is determined by multiplying fasting insulin with fasting plasma glucose and then dividing the result by 22.5 (29). All routine biochemical tests were executed in strict adherence to the standards specified in the NHANES Laboratory/Medical Technologist Procedure Manual.

### Calculation of AIP

AIP is derived from the ratio of fasting TG to HDL-C after undergoing logarithmic transformation, calculated as AIP = lg(TG/HDL-C) (11). The participants were categorized into four quartile groups according to their AIP values: Q1 (-1.25, -0.37), Q2 (-0.37, -0.15), Q3 (-0.15, 0.08), and Q4 (0.08, 0.85).

### MAFLD diagnosis

The diagnosis of MAFLD is conducted based on the 2020 consensus of the national expert panel. In brief, participants with hepatic steatosis who also exhibit any one of the following conditions: overweight/obesity, type 2 diabetes, or metabolic dysregulation, are diagnosed with MAFLD (1).

In this study, the existence of hepatic steatosis was confirmed using imaging methods. It is worth mentioning that the NHANES (2017–2018) survey employed a new method by combining ultrasound and vibration-controlled transient elastography (VCTE) to assess liver function. Qualified NHANES health professionals performed these evaluations on suitable participants at the NHANES Mobile Examination Center (MEC), utilizing FibroScan^®^ technology. This device measures ultrasonic attenuation, which correlates with hepatic steatosis, and documents controlled attenuation parameters (CAP). Previous studies have demonstrated the reliability of CAP in determining the presence of hepatic steatosis, with CAP values of 248 dB/m or higher indicating the presence of hepatic steatosis (30). In addition, we collected liver stiffness measurements(LSM) to assess liver fibrosis. The extent of fibrosis can be classified into three categories: F2, F3, and F4, with thresholds of 8.2, 9.7, and 13.6 kPa, respectively (31, 32).

### Statistical analysis

As recommended by the NHANES guidelines (33), we considered both complex sampling designs and sampling weights in the process of analyzing NHANES data. Because we used the index in fasting state (fasting TG) in this study, we chose fasting subsample MEC Weight (2017–2018). Basic characteristics were presented as counts and percentages (%) for categorical variables and as medians (interquartile range) for continuous variables. Chi-square test was used for differences between groups for categorical variables and Kruskal-Wallis test for differences between groups for continuous variables. Weighted logistic regression was employed to evaluate the relationship between AIP and MAFLD, resulting in the establishment of three models. Model 1 did not adjust for any confounding factors, Model 2 adjusted for sex and age, and Model 3 adjusted for race, education level, PIR, BMI, smoking status, drinking status, hyperlipidemia, hypertension, diabetes, physical activities status, ALT, AST, and lipid-lowering drugs on the basis of Model 2. Sensitivity analyses were performed using further matching propensity score (PSM) to assess whether the association between AIP and MAFLD was stable and reliable. A propensity score calculated for five demographic factors: sex, age, race, educational level, and PIR in the MAFLD and no MAFLD groups, matched 1 to 1 according to the score, with a caliper set at 0.01. Weighted multivariate logistic regression was performed on the data after PSM to assess whether the association between AIP and MAFLD remained significant. The dose-response relationship between AIP and MAFLD was visualized with RCS with three knots, and the predictive accuracy of AIP was assessed using ROC curves. All statistical analyses were performed using R (version 4.2.1, R Core Team 2020, Vienna, Austria) and MedCalc (version 20.022, MedCalc Software Ltd, Ostend, Belgium), considering a *P*-value less than 0.05 as statistically significant.

## Result

### Characteristics of participants

A total of 1090 participants were included in this study, including 556 males and 534 females. Participants were divided into Q1-Q4 groups according to AIP quartiles. Compared with the lowest quartile (Q1) group, participants with higher AIP were generally male, Mexican American, current smokers, hyperlipidemia, and diabetes patients, with higher BMI, waist circumference, fasting blood glucose, HOMA-IR, hs-CRP, TC, TG, LDL-C, ALT levels, and lower HDL-C levels(all *P*-values < 0.05), while there were no differences in age, drinking status, PIR, and AST (all *P*-values > 0.05), and the baseline characteristics of the participants are detailed in **Table 1**.

**Table 1:** Baseline characteristics of the study population based on AIP quartile grouping.

Variable	Total (N=1090)	Q1 (-1.25,-0.37)	Q2 (-0.37,-0.15)	Q3 (-0.15,0.08)	Q4 (0.08,0.85)	P value
Age (years)	44.00 (30.00,58.00)	38.00 (27.00,54.00)	41.00 (27.00,58.00)	47.00 (29.00,62.00)	47.00 (36.00,57.00)	0.06
Sex, n (%)						< 0.0001
Female	534 (49.06)	176 (64.19)	157 (53.35)	111 (42.91)	90 (34.46)	
Male	556 (50.94)	96 (35.81)	116 (46.65)	161 (57.09)	183 (65.54)	
RACE, n (%)						0.01
Non-Hispanic Black	239 (10.19)	91 (14.87)	70 (12.32)	57 (9.35)	21 (3.81)	
Mexican American	154 (8.99)	23 (5.15)	38 (8.50)	38 (9.39)	55 (13.29)	
Non-Hispanic White	390 (66.05)	88 (68.31)	93 (63.55)	102 (65.06)	107 (66.98)	
Other Race	307 (14.77)	70 (11.66)	72 (15.63)	75 (16.20)	90 (15.92)	
Education levels, n (%)						0.01
< high school	151 (7.22)	26 (3.73)	35 (7.06)	37 (6.71)	53 (11.75)	
= high school	266 (27.11)	60 (23.48)	56 (19.71)	80 (33.36)	70 (31.97)	
> high school	673 (65.66)	186 (72.79)	182 (73.23)	155 (59.94)	150 (56.28)	
Smoking status, n (%)						0.001
Never	657 (59.76)	190 (67.48)	173 (60.03)	162 (63.09)	132 (47.62)	
Former	239 (24.29)	59 (25.08)	58 (28.44)	54 (18.19)	68 (25.59)	
Current	194 (15.95)	23 (7.44)	42 (11.54)	56 (18.72)	73 (26.79)	
Drinking status, n (%)						0.19
Never	118 (7.16)	35 (4.60)	30 (8.68)	26 (10.37)	27 (5.25)	
Mild	483 (45.21)	117 (43.70)	128 (42.59)	122 (47.35)	116 (47.26)	
Moderate	236 (22.18)	74 (28.40)	54 (24.25)	57 (17.26)	51 (18.29)	
Heavy	253 (25.45)	46 (23.30)	61 (24.48)	67 (25.02)	79 (29.20)	
BMI (kg/m²)	27.80 (23.80,32.90)	24.30 (21.70,28.50)	27.30 (23.40,32.00)	29.20 (25.60,34.30)	30.70 (27.30,35.40)	< 0.0001
BMI category, n (%)						< 0.0001
Normal weight (< 25)	329 (31.55)	142 (55.83)	99 (35.60)	55 (19.97)	33 (12.64)	
Over weight (25–30)	326 (29.17)	66 (24.82)	77 (29.88)	89 (31.65)	94 (30.78)	
Obesity (≥ 30)	435 (39.27)	64 (19.35)	97 (34.53)	128 (48.38)	146 (56.59)	
Waist circumference, (cm)	97.40 (85.60,110.40)	86.10 (78.00,95.80)	94.50 (84.50,107.70)	101.20 (92.50,112.50)	105.30 (97.10,116.60)	< 0.0001
Hip circumference, (cm)	104.10 (97.70,114.60)	100.30 (94.20,106.80)	102.50 (97.30,116.10)	107.00 (101.00,116.00)	107.20 (101.20,118.80)	< 0.0001
Hyperlipidemia, n (%)	706 (62.48)	101 (33.12)	140 (48.20)	204 (76.23)	261 (94.70)	< 0.0001
DM,n (%)	186 (12.63)	17 (2.18)	38 (8.32)	52 (15.57)	79 (25.34)	< 0.0001
Hypertension, n (%)	413 (33.88)	71 (21.29)	89 (27.16)	126 (45.36)	127 (42.61)	0.002
MAFLD, n (%)	563 (50.50)	70 (23.02)	115 (46.34)	164 (61.90)	214 (73.27)	< 0.0001
LSM (kPa)	4.80 (4.00,6.10)	4.80 (4.00,5.80)	4.80 (3.90, 6.10)	4.70 (4.00, 6.10)	5.40 (4.20,6.20)	0.06
Lipid-lowering drugs, n (%)	168 (14.10)	31 (11.22)	31 (7.89)	50 (19.34)	56 (17.99)	0.05
Physical activities status, n (%)						0.03
< 150min/week	162 (13.52)	28 (6.18)	41 (16.61)	45 (18.95)	48 (13.10)	
≥ 150min/week	928(86.48)	244 (93.82)	232 (83.39)	227 (81.05)	225 (86.90)	
PIR, n (%)						0.24
< 1	177(10.97)	39 (8.10)	37 (8.16)	51 (14.23)	50 (13.56)	
1–3	451(33.54)	96 (28.01)	129 (36.88)	111 (35.87)	115 (34.01)	
> 3	462(55.49)	137 (63.89)	107 (54.96)	110 (49.90)	108 (52.43)	
Laboratory data						
HbA1c (%)	5.40(5.20,5.70)	5.30 (5.10,5.50)	5.40 (5.10,5.60)	5.40 (5.20,5.80)	5.50 (5.30,6.00)	< 0.0001
FBG (mmol/L)	5.66(5.33,6.11)	5.44 (5.16,5.77)	5.55 (5.27,6.00)	5.83 (5.38,6.27)	5.88 (5.55,6.55)	< 0.0001
HOMA-IR	2.21(1.37,4.05)	1.42 (0.88,1.95)	1.99 (1.26,3.19)	2.86 (1.77,4.25)	3.79 (2.25,6.57)	< 0.0001
hs-CRP (mg/L)	1.57(0.73,3.72)	0.83 (0.48,1.95)	1.64 (0.81,3.85)	2.09 (1.02,4.73)	2.32 (1.01,4.64)	0.002
TC (mmol/L)	4.65(4.11,5.40)	4.45 (3.96,5.04)	4.55 (4.06,5.28)	4.76 (4.14,5.46)	5.04 (4.37,5.79)	< 0.001
HDL-C (mmol/L)	1.37(1.14,1.68)	1.78 (1.55,1.99)	1.45 (1.24,1.71)	1.27 (1.16,1.45)	1.06 (0.96,1.19)	< 0.0001
TG (mmol/L)	0.95(0.63,1.48)	0.53 (0.43,0.63)	0.81 (0.67,0.93)	1.16 (0.99,1.39)	1.94 (1.66,2.35)	< 0.0001
LDL-C (mmol/L)	2.77(2.25,3.34)	2.38 (2.07,2.92)	2.77 (2.30,3.13)	2.97 (2.33,3.49)	3.08 (2.48,3.65)	< 0.001
ALT (U/L)	19.00(14.00,27.00)	16.00 (12.00,22.00)	17.00 (13.00,22.00)	20.00 (14.00,31.00)	24.00 (17.00,35.00)	< 0.001
AST (U/L)	20.00(16.00,24.00)	19.00 (17.00,24.00)	20.00 (16.00,22.00)	20.00 (16.00,24.00)	20.00 (17.00,26.00)	0.27

BMI, body mass index; DM, diabetes mellitus; MAFLD, metabolic associated fatty liver disease; PIR, poverty income ratio; LSM, liver stiffness measurement; FBG, fasting blood glucose; HOMA-IR, homeostasis model assessment of insulin resistance; hs-CRP, high-sensitivity c-reactive protein; TC, total cholesterol; HDL-C, high-density lipoprotein cholesterol; TG, triglycerides; LDL-C, low-density lipoprotein cholesterol; ALT, alanine aminotransferase; AST, aspartate aminotransferase.

### Association between AIP and the presence of MAFLD

The association between AIP and MAFLD prevalence was evaluated based on weighted logistic regression. In the logistic regression model without adjustment, the risk of MAFLD gradually increased in the quartiles with higher AIP compared with the lowest quartile (Q1) (*P* for trend < 0.0001). Multivariate logistic regression models(Model3) adjusting for potential confounders such as sex, age, race, education level, PIR, BMI, smoking status, drinking status, hyperlipidemia, hypertension, diabetes, physical activities status, ALT, AST, and lipid-lowering drugs, showed that the ORs for participants in Q2, Q3, and Q4 compared to those in Q1 based on their AIP were 2.00 (95% CI 1.03 to 3.91), 2.63 (95% CI 1.39 to 4.96), and 3.85 (95% CI 1.55 to 9.52), respectively (*P* for trend = 0.006). When AIP was treated as a continuous variable, a strong positive association between AIP and the prevalence of MAFLD remained (Model3: OR = 4.71, 95%CI 1.70–13.01), as detailed in **Table 2**. Subsequently, in order to further validate the stability of the results and reduce the impact of demographic factors on the results, we used PSM under weighted conditions to adjust demographic characteristics such as gender, age, race, educational level, and PIR between MAFLD patients and those without MAFLD, and the comparison of demographic data between the two groups after adjustment is detailed in **Supplementary Table 1**. The results of multivariate logistic regression after PSM still suggested a positive association between AIP and the prevalence of MAFLD (Model3: OR = 3.12, 95%CI 1.15–8.42), as detailed in **Table 3**.

**Table 2:** Association of AIP as a continuous variable and quartiles with MAFLD.

AIP	Number (%)	Model1		Model2		Model3	
	563	OR (95%CI)	*P*	OR (95%CI)	*P*	OR (95%CI)	*P*
as continuous variable	–	14.28 (8.14–25.06)	< 0.0001	14.06 (7.96–24.82)	< 0.0001	4.71 (1.70–13.01)	0.01
Q1 (-1.25,-0.37)	70 (12.43)	REF		REF		REF	
Q2 (-0.37,-0.15)	115 (20.43)	2.89 (1.55–5.38)	0.003	2.96 (1.57–5.60)	0.004	2.00 (1.03–3.91)	0.04
Q3 (-0.15,0.08)	164 (29.13)	5.43 (3.24–9.12)	< 0.0001	5.34 (3.13–9.10)	< 0.0001	2.63 (1.39–4.96)	0.01
Q4 (0.08,0.85)	214 (38.01)	9.17 (5.13–16.39)	< 0.0001	9.03 (4.75–17.17)	< 0.0001	3.85 (1.55–9.52)	0.01
*P* for trend		< 0.0001		< 0.0001		0.006	

Model 1 was the crude model; Model 2 was adjusted for sex and age; Model 3 was adjusted for sex, age, race, education level, PIR, BMI, smoking status, drinking status, hyperlipidemia, hypertension, DM, physical activities status, ALT, AST, and lipid-lowering drugs.

AIP, atherogenic index of plasma; MAFLD, metabolic associated fatty liver disease; OR, odds ratio; CI, confidence interval; PIR, poverty income ratio; BMI, body mass index; DM, diabetes mellitus; ALT, alanine aminotransferase; AST, aspartate aminotransferase.

**Table 3:** Association of AIP as a continuous variable and quartiles with MAFLD after PSM.

AIP	Number(%)	Model1		Model2		Model3	
	427	OR(95%CI)	*P*	OR(95%CI)	*P*	OR(95%CI)	*P*
as continuous variable	–	10.22(5.10–20.51)	< 0.0001	11.42(5.83–22.38)	< 0.0001	3.91(1.40–10.90)	0.01
Q1(-1.25,-0.36)	56(13.12)	REF		REF		REF	
Q2(-0.36,-0.16)	89(20.84)	2.71(1.30–5.64)	0.01	2.79(1.31–5.93)	0.01	1.80(0.83–3.92)	0.13
Q3(-0.16,0.07)	122(28.57)	4.70(2.43–9.09)	< 0.001	4.98(2.61–9.47)	< 0.001	3.02(1.49–6.08)	0.004
Q4(0.07,0.85)	160(37.47)	7.40(3.83–14.31)	< 0.0001	8.00(4.02–15.89)	< 0.0001	3.12(1.15–8.42)	0.03
*P* for trend		< 0.0001		< 0.0001		0.014	

Model 1 was the crude model; Model 2 was adjusted for sex and age; Model 3 was adjusted for sex, age, race, education level, PIR, BMI, smoking status, alcohol drinking, hyperlipidemia, hypertension, DM, physical activities status, ALT, AST, and lipid-lowering drugs.

AIP, atherogenic index of plasma; MAFLD, metabolic associated fatty liver disease; PSM, Propensity score matching; OR, odds ratio; CI, confidence interval.

In addition, we analyzed the association between AIP and the degree of liver fibrosis, but the results showed no significant association between AIP and liver fibrosis, as detailed in **Supplementary Table 2**.

### Dose-relationship between AIP and MAFLD

**Figure 2:**
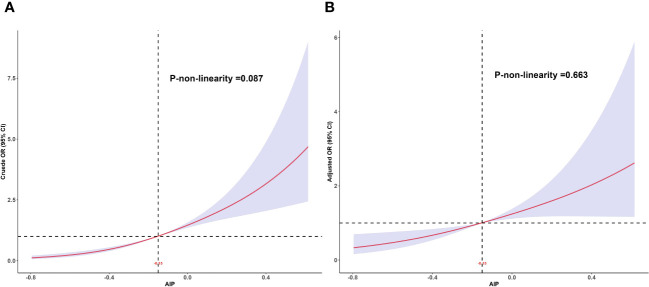
Dose-response relationship between AIP and MAFLD. **(A)** Unadjusted dose-response relationship between AIP and MAFLD; **(B)** Adjusted dose-response relationship between AIP and MAFLD. Adjusted factors include sex, age, race, education level, PIR, BMI, smoking status, drinking status, hyperlipidemia, hypertension, DM, physical activities status, ALT, AST, and lipid-lowering drugs. AIP, atherogenic index of plasma; MAFLD, metabolic associated fatty liver disease; PIR, poverty income ratio; BMI, body mass index; DM, diabetes mellitus; ALT, alanine aminotransferase; AST, aspartate aminotransferase.

The RCS was used to assess the dose-response relationship between AIP and MAFLD and to clarify the pattern of this dose-response relationship. The results showed a linear dose-response relationship between AIP and MAFLD (*P* for adjusted non-linearity = 0.663), whether adjusted for confounders or not, as shown in **Figure 2**.

**Figure 3:**
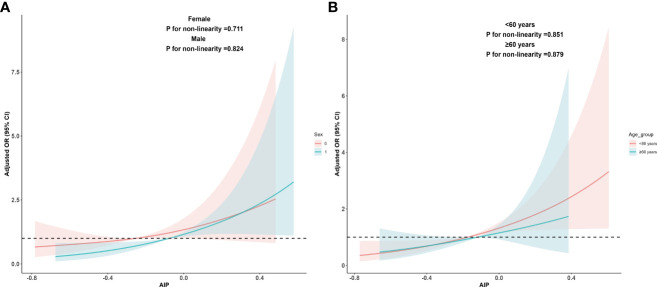
Dose-response relationship between AIP and MAFLD in sex and age subgroups. **(A)** Adjusted dose-response relationship between AIP and MAFLD in sex subgroups; **(B)** Adjusted dose-response relationship between AIP and MAFLD in age subgroups. Adjusted factors include sex, age, race, education level, PIR, BMI, smoking status, drinking status, hyperlipidemia, hypertension, DM, physical activities status, ALT, AST, and lipid-lowering drugs. When analyzing sex groups, sex factors should be excluded from confounding factors, and the same applies when analyzing age subgroups. AIP, atherogenic index of plasma; MAFLD, metabolic associated fatty liver disease; PIR, poverty income ratio; BMI, body mass index; DM, diabetes mellitus; ALT, alanine aminotransferase; AST, aspartate aminotransferase.

Due to significant differences in MAFLD prevalence among different age groups and genders, we re-evaluated the dose-response relationship between AIP and MAFLD within these subgroups. The results indicated that a linear dose-response relationship between AIP and MAFLD persisted in both age subgroups (<60 years: *P* for adjusted non-linearity = 0.851, ≥60 years: *P* for adjusted non-linearity = 0.879) and gender subgroups (Female: *P* for adjusted non-linearity = 0.711, Male: *P* for adjusted non-linearity = 0.824). These findings are detailed in **Figure 3**.

### Predictive value of AIP for the MAFLD

**Figure 4:**
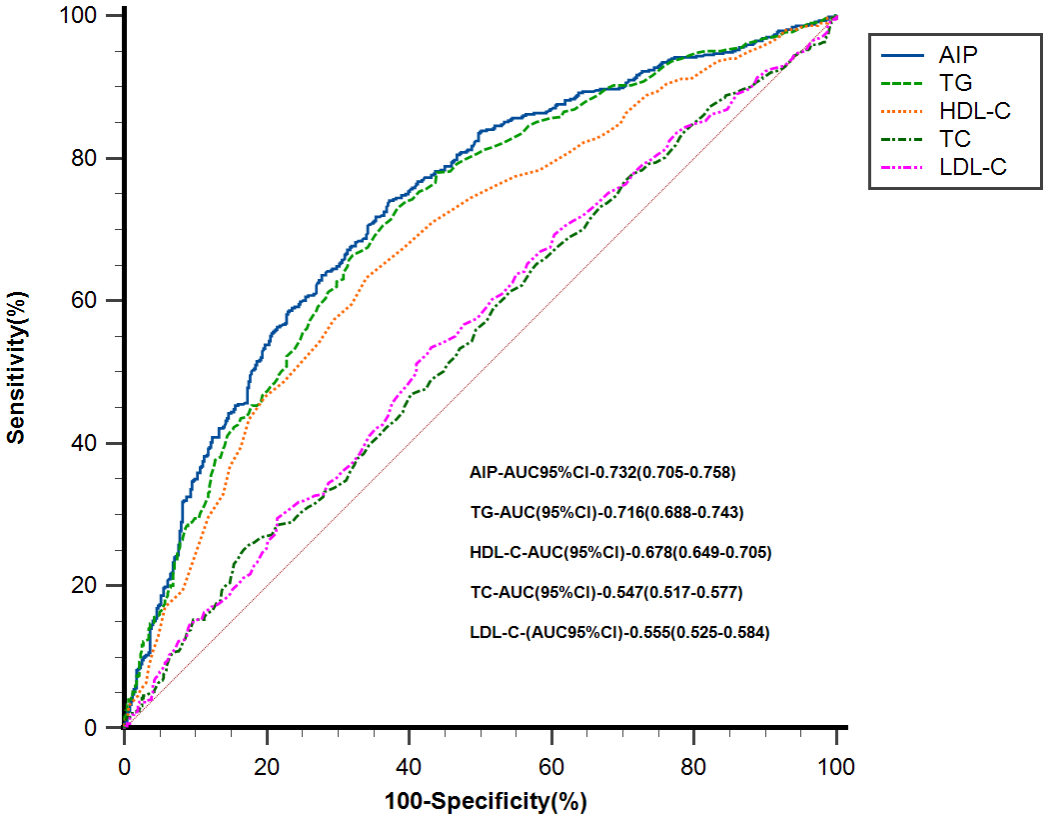
ROC analysis of AIP and traditional lipid parameters for predicting the risk of MAFLD. AIP, atherogenic index of plasma; MAFLD, metabolic associated fatty liver disease; TG, triglyceride; HDL-C, high-density lipoprotein cholesterol; TC, total cholesterol; LDL-C, low-density lipoprotein cholesterol; CI, confidence interval.

ROC curves were used to evaluate the value of AIP and traditional lipid parameters (TG, HDL-C, TC, LDL-C) in predicting the risk of MAFLD. The results showed that AIP(AUC = 0.732, 95%CI 0.705–0.758) predicted the risk of MAFLD better than individual lipid parameters, as shown in **Figure 4**. The optimal cut-off level for AIP was -0.21 (sensitivity = 74.07%, specificity = 62.81%). Differences between AIP and traditional lipid parameters in predicting the risk of MAFLD are detailed in **Supplementary Table 3**.

## Discussion

In this cross-sectional analysis of NHANES 2017–2018, we found a strong association between AIP and MAFLD prevalence. Our study demonstrates for the first time that there is a linear dose-response pattern between AIP and MAFLD, that is, as AIP increases, so does the risk of MAFLD. Finally, the ROC curves analysis demonstrated that AIP is superior to traditional lipid markers in predicting the risk of MAFLD.

Similar to NAFLD, hepatic steatosis is also one of the key features of MAFLD. However, previous studies have demonstrated that individual lipid parameters lack specificity in identifying NAFLD (34, 35), and there remains controversy regarding the evidence of the role of individual lipids in promoting hepatic fat accumulation (7, 36). The advantage of AIP compared with traditional blood lipid indicators is that it combines two blood lipid indicators, TG and HDL-C, in the form of ratio, which can reflect more information about blood lipid metabolism and is more stable than a single blood lipid index. TG is one of the common indicators of atherosclerosis, especially in recent studies, which have found that TG-rich lipoprotein cholesterol plays a significant role in increasing residual cardiovascular risk (37). In contrast, HDL-C is considered to have anti-inflammatory and anti-atherosclerotic effects (38–40). Therefore, to some extent, the AIP can be regarded as an indicator reflecting the balance between atherogenesis and anti-atherogenesis in the body. Previous studies have suggested that MAFLD may mediate the development and progression of atherosclerosis (41, 42). Therefore, the strong association between AIP and MAFLD may be related to their common involvement in the process of atherosclerosis in the body. In addition, IR also plays an important role in hepatic steatosis. When IR occurs in the body, on the one hand, it leads to accumulation of free fatty acids in liver tissue and increases lipid synthesis (43); on the other hand, it also leads to reduced adiponectin availability and weakens its ability to regulate fat metabolism. At the same time, reduced availability of adiponectin further aggravates body IR, causing a vicious cycle (2, 44). Previous studies have already confirmed a close association between AIP and IR (17, 45). From this, it can be inferred that the association between AIP and MAFLD may be explained based on the strong associations between AIP and atherosclerosis as well as IR. However, further studies are still needed to validate these hypotheses. Shin et al. (21) found that AIP was associated with obesity index, blood glucose, and lipid profile in Korean men in a study based on data from the Korea National Health and Nutrition Examination Survey (KNHANES) from 2013 to 2014, and these real-world evidence also directly indicated that AIP was closely related to multiple metabolic indexes of the body, not only to lipids.

To the best of our knowledge, this is the first study to investigate the dose-response relationship between AIP and MAFLD in the adult population of the United States. Although several previous studies have described the association between AIP and MAFLD, none of these studies explored the dose-response relationship between AIP and MAFLD, and the emphasis of these studies was different. A cross-sectional analysis of 864 Chinese participants by Duan et al. showed a positive association between AIP and MAFLD, and combined AIP with BMI and waist circumference to construct a new index that can improve the predictive ability of MAFLD (22). Samimi et al. showed AIP to be a valid predictor of MAFLD in patients with type 2 diabetes, they were more concerned about the relationship between AIP and MAFLD in patients with type 2 diabetes mellitus (T2DM) and this study was mainly conducted in Iranian population (23). Recently, Wang et al. explored the association between AIP and MAFLD on the basis of NHANES survey data (46). Similar to the two previous studies, this study did not explore the dose-response relationship between AIP and MAFLD. Furthermore, when constructing the final model, the study did not consider the impact of exercise and alcohol consumption on the outcome. In our study, we diagnosed MAFLD according to the recommendations of the 2020 international expert panel consensus (1). However, unlike Wang et al.’s study, which utilized a diagnostic threshold of CAP ≥ 285 dB/m for hepatic steatosis, we employed a lower threshold of CAP ≥ 248 dB/m (30), which could identify more underlying patients with MAFLD (34, 47). In addition, to avoid the effect of diet on TG as much as possible, we used TG measured in the fasting state. Considering the significant sex and age differences in the prevalence of MAFLD (48, 49), we explored whether the linear dose-response relationship between AIP and MAFLD remains in the sex and age subgroups separately, as well as the specific pattern of dose-response relationship in the sex and age subgroups. The results showed that the linear dose-response relationship between AIP and MAFLD remained stable across sex and age subgroups.

It has been shown that MAFLD increases the risk of acute myocardial infarction (AMI) and stroke by 35% and 26%, respectively (50), and the results of Chung et al. suggest that MAFLD with DM can be a strong predictor of all-cause mortality and disease-specific mortality (51). It is therefore highly desirable to identify underlying MAFLD patients in clinical work. However, limited by the actual situation of clinical work, it is difficult for clinicians to conduct universal screening of patients with MAFLD. Our results suggest a strong positive association between AIP and MAFLD, and AIP is superior to traditional lipid parameters in predicting the prevalence of MAFLD. Therefore, clinicians may be able to raise concerns about patients with abnormally elevated AIP and consider MAFLD-related diagnostic tests if necessary.

This study has the following limitations: 1. Based on the design of a cross-sectional study, this study cannot identify whether there is a causal association between AIP and MAFLD; 2. The conclusions of this study apply only to the US adult population; 3. The conclusions of this study warrant further validation in a larger cohort study.

## Conclusion

The findings of this study revealed a robust positive association between AIP and MAFLD. This association proved significant, irrespective of whether AIP was treated as a continuous variable or categorized using quartiles. At the same time, potential linear dose-response relationships between AIP and MAFLD have been elucidated, that is, the risk of MAFLD also increased as the AIP value increased. Given that MAFLD significantly increases the risk of adverse outcomes for patients, particularly in terms of cardiovascular and cerebrovascular diseases, physicians may consider using AIP to screen for MAFLD in order to better assess the underlying risks for these patients. However, further validation of this conclusion is warranted through larger-scale cohort studies.

## Data availability statement

The original contributions presented in the study are included in the article/**Supplementary material**. Further inquiries can be directed to the corresponding author.

## Ethics statement

The studies involving humans were approved by National Center for Health Statistics Research Ethics Review Board (Protocol number: 2018-01). The studies were conducted in accordance with the local legislation and institutional requirements. The participants provided their written informed consent to participate in this study.

## Author contributions

YC: Conceptualization, Writing – original draft, Writing – review & editing. CL: Data curation, Writing – review & editing. HJ: Data curation, Writing – review & editing. QZ: Writing – review & editing. XZ: Supervision, Writing – review & editing.
