# Supplementary material for: Elevated AIP is associated with the prevalence of MAFLD in the US adults: evidence from NHANES 2017–2018
Source: Front Endocrinol (Lausanne). 2024 May 14;15:1405828. doi: 10.3389/fendo.2024.1405828 (PMC11130487; doi:10.3389/fendo.2024.1405828)
Supplement: Supplementary file 1 [file Table_1.docx]

**Supplementary Table 1 Comparison of demographic characteristics between the two groups after PSM**

| **Variable** | **Without MAFLD(N=427)** | **MAFLD(N=427)** | ***P*** |
| --- | --- | --- | --- |
| Age(years) | 43.00(29.00,55.00) | 46.00(33.00,59.00) | 0.22 |
| Sex,n(%) |  |  | 0.68 |
| Female | 208(50.54) | 207(49.33) |  |
| Male | 219(49.46) | 220(50.67) |  |
| RACE,n(%) |  |  | 0.14 |
| Non-Hispanic Black | 117(13.01) | 79( 8.83) |  |
| Mexican American | 45( 7.24) | 73(10.72) |  |
| Non-Hispanic White | 136(65.76) | 158(64.65) |  |
| Other Race | 129(14.00) | 117(15.80) |  |
| Education levels |  |  | 0.93 |
| <high school | 64(7.81) | 57(8.14) |  |
| =high school | 99(25.95) | 115(26.73) |  |
| >high school | 264(66.24) | 255(65.13) |  |
| PIR |  |  | 0.93 |
| ≤1 | 79(11.82) | 67(11.25) |  |
| 1-3 | 169(33.63) | 193(35.30) |  |
| >3 | 179(54.55) | 167(53.45) |  |

Abbreviations: PSM, propensity score matching; MAFLD, metabolic associated fatty liver disease; PIR, poverty income ratio.
