# Supplementary material for: Elevated AIP is associated with the prevalence of MAFLD in the US adults: evidence from NHANES 2017–2018
Source: Front Endocrinol (Lausanne). 2024 May 14;15:1405828. doi: 10.3389/fendo.2024.1405828 (PMC11130487; doi:10.3389/fendo.2024.1405828)
Supplement: Supplementary file 2 [file Table_2.docx]

**Supplementary Table 2 Association of AIP as a continuous variable and quartiles with the degree of fibrosis**

| **AIP** | **Model1** |  |  | **Model2** |  |  | **Model3** |  |
| --- | --- | --- | --- | --- | --- | --- | --- | --- |
|  | **OR(95%CI)** | ***P*** |  | **OR(95%CI)** | ***P*** |  | **OR(95%CI)** | ***P*** |
| **F2 (LSM ≥ 8.2kPa)** |  |  |  |  |  |  |  |  |
| as continuous variable | 1.87(0.65-5.34) | 0.22 |  | 1.78(0.51-6.22) | 0.34 |  | 0.49(0.12-2.03) | 0.30 |
| Q1(-1.25,-0.37) | REF |  |  | REF |  |  | REF |  |
| Q2(-0.37,-0.15) | 1.87(0.94-3.71) | 0.07 |  | 1.81(0.81-4.04) | 0.13 |  | 1.33(0.67-2.65) | 0.39 |
| Q3(-0.15,0.08) | 2.26(0.62-8.22) | 0.20 |  | 1.99(0.43-9.22) | 0.34 |  | 0.75(0.30-1.83) | 0.50 |
| Q4(0.08,0.85) | 1.64(0.46-5.79) | 0.41 |  | 1.51(0.35-6.56) | 0.55 |  | 0.36(0.10-1.32) | 0.11 |
| *P* for trend | 0.415 |  |  | 0.591 |  |  | 0.056 |  |
| **F3 (LSM ≥ 9.7kPa)** |  |  |  |  |  |  |  |  |
| as continuous variable | 1.98(0.52-7.57) | 0.29 |  | 1.81(0.30-11.04) | 0.49 |  | 0.96(0.24-3.90) | 0.95 |
| Q1(-1.25,-0.37) | REF |  |  | REF |  |  | REF |  |
| Q2(-0.37,-0.15) | 1.96(0.64-6.02) | 0.21 |  | 1.86(0.49-7.00) | 0.32 |  | 2.28(0.84-6.19) | 0.10 |
| Q3(-0.15,0.08) | 2.51(0.38-16.63) | 0.31 |  | 2.11(0.21-21.40) | 0.49 |  | 1.74(0.58-5.26) | 0.30 |
| Q4(0.08,0.85) | 1.98(0.34-11.63) | 0.42 |  | 1.76(0.19-16.11) | 0.58 |  | 0.96(0.32-2.83) | 0.93 |
| *P* for trend | 0.415 |  |  | 0.614 |  |  | 0.491 |  |
| **F4 (LSM ≥ 13.6kPa)** |  |  |  |  |  |  |  |  |
| as continuous variable | 1.25(0.20-7.86) | 0.80 |  | 1.27(0.10-16.52) | 0.85 |  | 0.93(0.16-5.48) | 0.93 |
| Q1(-1.25,-0.37) | REF |  |  | REF |  |  | REF |  |
| Q2(-0.37,-0.15) | 0.75(0.08-7.24) | 0.78 |  | 0.72(0.06-9.06) | 0.78 |  | 0.29(0.05-1.91) | 0.18 |
| Q3(-0.15,0.08) | 1.77(0.18-17.31) | 0.59 |  | 1.60(0.10-25.59) | 0.71 |  | 0.86(0.18-4.21) | 0.84 |
| Q4(0.08,0.85) | 0.89(0.11-7.24) | 0.91 |  | 0.87(0.06-12.66) | 0.91 |  | 0.32(0.06-1.77) | 0.18 |
| *P* for trend | 0.838 |  |  | 0.910 |  |  | 0.306 |  |
| **F (LSM ≥ 8.5kPa)** |  |  |  |  |  |  |  |  |
| as continuous variable | 1.89(0.67-5.32) | 0.21 |  | 1.88(0.51-6.96) | 0.31 |  | 0.67(0.14-3.22) | 0.59 |
| Q1(-1.25,-0.37) | REF |  |  | REF |  |  | REF |  |
| Q2(-0.37,-0.15) | 2.00(0.85-4.69) | 0.10 |  | 1.95(0.70-5.46) | 0.18 |  | 1.68(0.68-4.16) | 0.24 |
| Q3(-0.15,0.08) | 2.55(0.58-11.27) | 0.19 |  | 2.27(0.38-13.63) | 0.33 |  | 1.04(0.37-2.96) | 0.93 |
| Q4(0.08,0.85) | 1.76(0.45-6.88) | 0.39 |  | 1.67(0.32-8.63) | 0.50 |  | 0.50(0.13-1.88) | 0.28 |
| *P* for trend | 0.367 |  |  | 0.523 |  |  | 0.121 |  |

**Note:** Model 1 was the crude model; Model 2 was adjusted for sex and age; Model 3 was adjusted for sex, age, race, education level, PIR, BMI, smoking status, drinking status, hyperlipidemia, hypertension, DM, physical activities status, ALT, AST, and lipid-lowering drugs. LSM ≥ 8.2, ≥ 9.7 and ≥ 13.6 kPa represent significant fibrosis (≥F2), advanced fibrosis (≥F3), and cirrhosis (F4), respectively.

**Abbreviations:** AIP, atherogenic index of plasma; MAFLD, metabolic associated fatty liver disease; OR, odds ratio; CI, confidence interval; LSM, liver stiffness measurement; PIR, poverty income ratio; BMI, body mass index; DM,diabetes mellitus; ALT, alanine aminotransferase; AST, aspartate aminotransferase.
