# Supplementary material for: Elevated AIP is associated with the prevalence of MAFLD in the US adults: evidence from NHANES 2017–2018
Source: Front Endocrinol (Lausanne). 2024 May 14;15:1405828. doi: 10.3389/fendo.2024.1405828 (PMC11130487; doi:10.3389/fendo.2024.1405828)
Supplement: Supplementary file 3 [file Table_3.docx]

**Supplementary Table3** **Performance of AIP and traditional lipid parameters in predicting the risk of MAFLD**

| **Variables** | **AUC** | **95%CI** | **Sensitivity** | **Specificity** | **Optiaml Cut-off** | ***P*-value** |
| --- | --- | --- | --- | --- | --- | --- |
| AIP | 0.732 | 0.705,0.758 | 74.07% | 62.81% | -0.21 | <0.0001 |
| TG | 0.716 | 0.688,0.743 | 73.00% | 61.67% | 0.86 | <0.0001 |
| HDL-C | 0.678 | 0.649,0.705 | 63.23% | 66.03% | 1.34 | <0.0001 |
| TC | 0.547 | 0.517,0.577 | 25.04% | 83.30% | 5.56 | 0.0069 |
| LDL-C | 0.555 | 0.525,0.584 | 53.46% | 56.93% | 3.80 | 0.0017 |

**Abbreviations:** AIP, atherogenic index of plasma; MAFLD, metabolic associated fatty liver disease; TG, triglyceride; HDL-C, high-density lipoprotein cholesterol; TC, total cholesterol; LDL-C, low-density lipoprotein cholesterol; CI, confidence interval.
